# Comparison of the ocular ultrasonic and optical biometry devices in the different quality measurements

**DOI:** 10.1016/j.optom.2023.05.001

**Published:** 2023-08-09

**Authors:** Masoud Khorrami-Nejad, Ahmed Mohammed Khodair, Mehdi Khodaparast, Farshid Babapour Mofrad, Farzaneh Dehghanian Nasrabadi

**Affiliations:** a Optometry Department, School of Rehabilitation, Tehran University of Medical Sciences, Tehran, Iran; b Translational Ophthalmology Research Center, Farabi Eye Hospital, Tehran University of Medical Sciences, Tehran, Iran; c Department of Medical Radiation Engineering, Science and Research Branch, Islamic Azad University, Tehran, Iran; d Optometry Department, School of Rehabilitation, Iran University of Medical Sciences, Tehran, Iran

**Keywords:** Optical biometer, Ultrasound biometer, Axial length, Anterior chamber depth, Lens thickness

## Abstract

**Purpose:**

To compare the reliability and agreement of axial length (AL), anterior chamber depth (ACD), and lens thickness (LT) measurements obtained with optical biometry based on swept-source optical coherence tomography (IOLMaster 700; Carl Zeiss, Germany) and an ultrasound biometry device (Nidek; US-4000 Echoscan, Japan) in different qualities of AL measurement.

**Methods:**

A total of 239 consecutive eyes of 239 cataract surgery candidates with a mean age of 56 ± 14 years were included. The quality measurements were grouped according to the quartiles of SD of the measured AL by IOLMaster 700. The first and fourth quartile's SD are defined as high and low-quality measurement, respectively, and the second and third quartiles’ SD is defined as moderate-quality.

**Results:**

The reliability of AL and ACD between the two devices in all patients and in different quality measurement groups was excellent with highly statistically significant (AL: all ICC=0.999 and *P*<0.001, ACD: all ICC>0.920 and *P*<0.001). AL and ACD in all quality measurements showed a very strong correlation between devices with highly statistically significant. However, there was poor (ICC=0.305), moderate (ICC=0.742), and good (ICC=0.843) reliability in measuring LT in low-, moderate-, and high-quality measurements, respectively. LT showed a very strong correlation (*r* = 0.854) with highly statistically significant (*P*<0.001) between devices only in patients with high-quality measurements.

**Conclusions:**

AL and ACD of the IOLMaster700 had outstanding agreements with the US-4000 ultrasound in different quality measurements of AL and can be used interchangeably. But LT should be used interchangeably cautiously only in the high-quality measurements group.

## Introduction

Intraocular lenses (IOL) power calculation is one of the main parts of the preoperative examination of cataractous patients.^1^ In order to determine accurate IOL power, some ocular parameters should be measured through biometry devices. The most important ocular biometry parameters include the axial length (AL), the corneal curvature (K-reading), corneal diameter (white-to-white), and the anterior chamber depth (ACD).^2^^,^^3^

There are two different technologies for performing ocular biometry measurements; ultrasonography and optical biometry.^4^, ^5^, ^6^, ^7^ Ophthalmic ultrasound is an easily available, cost-effective and reliable imaging method for measuring oculometric parameters.^8^ A-mode applanation ultrasound is a contact biometry method in which the device's probe is placed directly on the corneal surface.^9^ The primary problem with the contact method is the unwanted pressure applied to the cornea during the test and can underestimate the measured AL and ACD.

In 1999, the German Carl Zeiss company introduced a non-contact optical-based device called IOLMaster.^10^ This device performs oculometric measurements such as AL, ACD, corneal curvature, and horizontal white-to-white (WTW) diameter.^11^ The incorporated technology in IOLMaster for measuring oculometric parameters is known as partial laser interference, which from the biometrics perspective, is called partial coherence interferometry (PCI).^12^ This system is approximately five times more accurate than applanation A-mode applanation US.^10^^,^ ^13^ Therefore, the distance between the corneal vertex and the retina is measured as fine as 0.02 mm, while the A-mode ultrasound scan has a measurement resolution of 0.1 to 0.12 mm.^14^ However, optical devices have some challenges when performing measurements in some patients with severe posterior subcapsular cataract, hypermature cataract, vitreous hemorrhage, and corneal opacity, that is due to the potential inability of the laser light to penetrate to the densely opaque media.^15^^,^^16^

In recent years, new biometric instruments based on the swept-source optical coherence tomography (SS-OCT) technology have been a non-invasive, high-sensitivity, and high-resolution medical imaging technology.^17^ The SS-OCT has a higher signal-to-noise ratio (SNR) than previous generations of OCT.^18^ Currently, IOLMaster 700 (Carl Zeiss Meditec AG, Jena, Germany) is an optical biometer that incorporates SS-OCT technology with the following specifications: the wavelength of the swept-frequency light source is 1055 nm, the scanning speed is 2000 times/second, and the scanning depth is 44 mm.^11^ The width of the anterior segment and retina covered by this device is 6 mm and 1 mm, respectively, with a tissue resolution of 22 μm.^19^ AL measurement results with the previous generation of IOLMaster could be easily evaluated by numerical values ​​called the reliability factor with SNR of the measured waveform.^20^^,^^21^ However, even if the reliability is high with an SNR of 2 or higher, the measurements could ​​vary, and the evaluation by the SNR might be questionable.^22^ The IOLMaster 700 represents the SD index to assure its metrics measurements instead of SNR value.^23^^,^^24^ In addition, the IOL-Master 700 requires the fovea to be observed on fundus image to confirm the correct fixation and the accuracy of the AL measurement.^11^^,^^25^

Previous studies have evaluated the agreement of eye measurements using some ultrasound devices with optical biometers.^26^, ^27^, ^28^ IOLMaster700, as an optical biometer tool, is very expensive due to the use of the advanced swept-source ocular coherence tomography imaging system, and it is not possible to provide it for many clinics and hospitals.^29^ On the other hand, the Echoscan US-4000 (Nidek Inc, Japan) is much more economical due to the incorporation of an ultrasound system and is used or may be installed in many clinics.^30^ The main purpose of the present study is to compare the agreement values between an advanced optical-based biometry machine (IOLMaster 700) and an ocular ultrasound-based biometry device (Echoscan US-4000) for AL, ACD, and lens thickness (LT) measurements in the groups of patients with different quality measurements. To our knowledge, this is the first study that will compare the ocular ultrasonic and optical biometry devices in different quality measurements.

## Patients and methods

This prospective consecutive case series study was performed on 239 candidates for cataract surgery at Farabi Eye Hospital, affiliated with the Tehran University of Medical Sciences, Tehran, Iran. The Tehran University of medical sciences' ethical committee approved the study, and all processes used adhered to the tenets of the Declaration of Helsinki, and these tenets were followed during all phases of examinations. After a verbal explanation of the aim of the study and the methods that were used on the potential subjects and their parents, written informed consent was obtained from all the patients included in the study.

Routine ophthalmic examinations were performed for all participants. The refractive error was measured using autorefraction (Topcon KR-800, Topcon Corporation, Tokyo, Japan), and the results were confirmed by Heine beta 200 retinoscope (Heine Optotechnik, Herrsching, Germany). Snellen E chart was used for measuring visual acuity, and the results were converted into logMAR.

### Biometry and procedures

1. ***Ocular optical biometry:*** In the present study, optical biometry was performed using IOLMaster700 (Carl Zeiss Meditec AG, Jena, Germany). IOLMaster 700 warns the low-quality measurements when the SD values record AL >0.027 mm, LT >0.038 mm, and ACD >0.021 mm.^11^ As there was no classification for checking the measurement quality by IOL Master 700, the following classification was used in this study; the quality measurements were grouped into low, moderate, and high-quality measurements according to the quartiles of SD of the AL. Each quartile comprises 25% of the SD of the measured AL by IOL Master 700. The first quartile SD is defined as a high-quality measurement, the second and third quartile SD is defined as a moderate-quality measurement, and then, the SD of the AL in the fourth quartile is defined as a low-quality measurement. The accuracy of the results for each group was determined independently. All optical biometry measurements were performed by the same experienced optometrist. Only patients with green indicators in all measured parameters were selected.
2. ***Ocular ultrasound biometry:*** The present study was performed with A-mode applanation ultrasound through the Echoscan US-4000 (Nidek Inc, Japan). According to the specifications provided by the manufacturer, this device has a 10 MHz solid probe and can measure AL, ACD, LT, and vitreous chamber length. The measurement accuracy is ± 0.1 mm, with a range of 12 to 40 mm. In addition, the gain and time gain compensation (TGC) ranges are 0 to 90 dB and 0 to −20 dB, respectively. Following instilling tetracaine eye drops, the probe was placed directly on the corneal center to measure biometric parameters. The measurement was repeated ten times, and the average of them was calculated by the device and was considered as the amount of AL, ACD, and LT. All ultrasound biometry measurements were done at the final part of the examination to prevent its effect on the ocular optical biometry measurements because of the possible changes in the tear film and epithelial layer as well as its corneal flattening effects.^31^ Controlling the patient's fixation during the examination is challenging for the examiner, and the direct contact of the probe with the cornea could affect the AL measurements.^32^, ^33^, ^34^

First optical biometry was performed by an optometrist, and after that, ocular ultrasound biometry measurements were performed by another expert optometrist who was blind to the results of optical biometry. All examinations were recorded between 09:00 and 13:00 to minimize the possible effect of diurnal fluctuations.

In our study, the classification was performed based on the measured AL using IOLMaster 700, but the ultrasound measurement considers as the baseline.

### Statistical analysis

In the present study, the correlation, reliability, and agreement of AL and ACD with ultrasound and optical biometry techniques in different quality measurements were analyzed. The collected data was analyzed using SPSS-24 software (IBM, Armonk, NY). Mean ± SD of AL, ACD, and LT in different study groups were reported. The intraclass correlation coefficient (ICC) was calculated to evaluate the reproducibility between the AL, ACD, and LT measurements of the two devices. Regression analysis was performed to determine a model of the mathematical relationship (the conversion factor) for AL between optical and ultrasound biometry techniques. Bland–Altman plots were applied to describe the agreement in different parameters between devices with 95% confidence intervals. ICC for reliability and Bland-Altman plots for the agreement between the mean findings of each device were analyzed. The first quartile SD is defined as a high-quality measurement, the second and third quartiles SD is defined as a moderate-quality measurement, and finally, the SD of the AL in the fourth quartile defines as a low-quality measurement. Stratification was accomplished to control AL. The confounding effects of age and gender were included in the regression and removed by considering these factors as covariates. A P-value less than 0.05 was considered statistically significant. A preliminary study on 15 cases showed that the mean AL difference was 0.046, and the SD of the AL differences was 0.155. The maximum allowed difference between the methods was considered 0.4 mm. We entered 0.046, 0.155 and 0.4 mm for the expected mean of AL differences, expected SD of differences and the maximum allowed difference between the two methods, respectively. α-level and β-level selected 0.05 and 0.20 (power is 80%), respectively. The minimum required number of pairs was calculated 223. Sample size calculation was done using MedCalc statistical software version 20.026.

## Results

This study was performed on 239 eyes [121 (48.8%) right eyes and 127 (51.2%) left eyes] of 239 patients (129 male and 110 female), with a mean age of 56 ± 14 years (ranges between 15 and 95 years). The mean value of best-corrected distance visual acuity (CDVA) was 0.61 ± 0.42 logMAR (ranges between 2.3 and 0.0 logMAR). Only in 6 (2.4%) patients, the CDVA was 2.3 logMAR (equal to the perception of hand movement or 20/4000). There was a weak correlation between the CDVA and SD of measured AL (*r* = 0.398, *P*<0.001). The mean spherical equivalent value was −1.62 ± 4.97D (ranges between −22.00 to +5.25D). One hundred and ten patients had poor retinoscopic reflex for measuring the refractive error.

The comparison of AL, ACD and LT in patients with different measurement qualities of AL is reported in Table 1. This table shows a significant difference in AL and LT measured by ultrasound biometer (Echoscan US-4000) in different measurement quality of AL.

1. ***The comparison of axial length between the two devices:***

**Table 1:** The comparison of axial length, anterior chamber depth, and lens thickness in patients with different measurement qualities of axial length.

			N	Mean	SD	Std. Error	95% Confidence Interval for Mean		Minimum	Maximum	*P*-value
							Lower Bound	Upper Bound			
**IOLMaster700 (Carl Zeiss AG, Germany)**	Axial length	Low-quality	60	24.22	2.65	0.34	23.53	24.90	21.15	34.47	0.388*
		Moderate quality	119	23.79	1.80	0.16	23.46	24.12	20.46	31.35	
		High-quality	60	23.26	1.21	0.16	22.95	23.57	20.63	27.12	
		Total	239	23.76	1.95	0.13	23.52	24.01	20.46	34.47	
	ACD	Low-quality	60	3.21	0.45	0.06	3.09	3.33	2.22	4.20	0.272⁎⁎
		Moderate quality	119	3.32	0.43	0.04	3.24	3.39	2.41	4.72	
		High-quality	60	3.25	0.42	0.05	3.14	3.36	2.01	4.17	
		Total	239	3.27	0.43	0.03	3.22	3.33	2.01	4.72	
	Lens thickness	Low-quality	60	4.35	0.60	0.08	4.20	4.51	3.10	6.20	0.791*
		Moderate quality	119	4.29	0.48	0.04	4.20	4.38	2.41	5.20	
		High-quality	60	4.32	0.43	0.06	4.21	4.44	3.36	5.50	
		Total	239	4.31	0.50	0.03	4.25	4.38	2.41	6.20	
**US-4000 Nidek Echo Scan**	Axial length	Low-quality	60	24.18	2.63	0.34	23.50	24.86	21.05	33.92	0.261*
		Moderate quality	119	23.74	1.80	0.16	23.42	24.07	20.37	31.30	
		High-quality	60	23.23	1.19	0.15	22.92	23.53	20.61	27.02	
		Total	239	23.72	1.94	0.13	23.48	23.97	20.37	33.92	
	ACD	Low-quality	60	3.34	0.41	0.05	3.24	3.45	2.53	4.30	0.249*
		Moderate quality	119	3.45	0.38	0.03	3.38	3.52	2.63	4.83	
		High-quality	60	3.39	0.40	0.05	3.29	3.49	2.28	4.21	
		Total	239	3.41	0.39	0.03	3.36	3.46	2.28	4.83	
	Lens thickness	Low-quality	60	3.94	0.67	0.09	3.76	4.11	1.89	5.21	0.018*
		Moderate quality	119	4.14	0.59	0.05	4.03	4.24	2.36	5.32	
		High-quality	60	4.28	0.51	0.07	4.15	4.41	2.89	5.61	
		Total	239	4.12	0.60	0.04	4.05	4.20	1.89	5.61	

⁎ Kruskal Wallis Test.

⁎⁎ One-way ANOVA. ACD, anterior chamber depth.

**Fig. 1:**
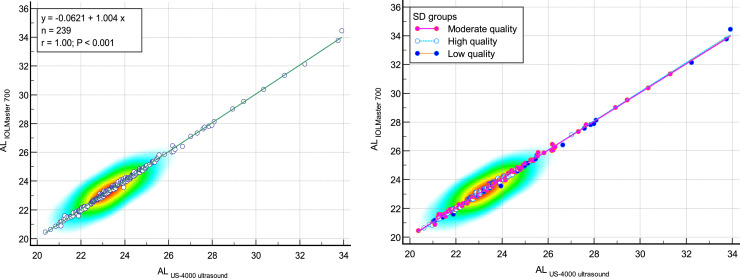
Scatter plots to compare mean values of axial length measured with IOLMaster 700 optical biometer and US-4000 ultrasound biometer among all patients (left), and patients classified by the quality of measurements (right). The solid line shows the regression line.

**Fig. 2:**
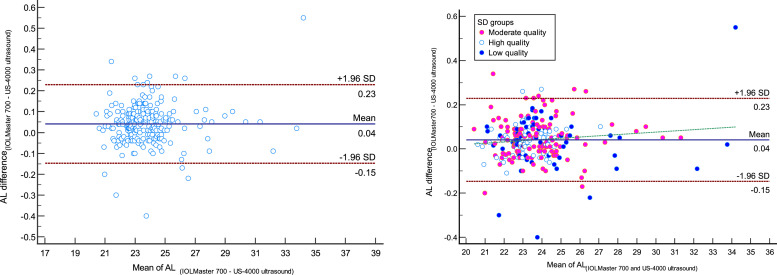
Bland–Altman plot for axial length (AL) comparing IOLMaster 700 optical biometer (Carl Zeiss Meditec AG, Jena, Germany) vs Echoscan US-4000 ultrasound biometer (Nidek Inc, Japan) among all patients (left), and patients classified by the quality of measurements (right). The dashed green line shows the regression line.

The comparison of measured AL with IOLMaster700 optical biometer and Echoscan US-4000 ultrasound biometer in different measurement qualities of AL are reported in Table 2. Scatter plots to compare mean values of AL measured with IOLMaster 700 optical biometer and Echoscan US-4000 ultrasound biometer among all patients are presented in Fig. 1. Also, Bland–Altman plots for AL comparing IOLMaster 700 optical biometer versus Echoscan US-4000 ultrasound biometer among all patients are shown in Fig. 2**.** The mean age of 11 patients with AL difference>0.2 mm was significantly higher than 228 patients with AL difference<0.2 mm (70 ± 14 vs. 58 ± 13, *P*<0.001).

2. ***The comparison of ACD between two devices:***

**Table 2:** The comparison of measured axial length with IOLMaster 700 optical biometer (Carl Zeiss Meditec AG, Jena, Germany) and Echoscan US-4000 ultrasound biometer (Nidek Inc, Japan) in different measurement qualities of axial length.

	Measurement quality	Devices	N	Mean	Std. Deviation	95% Confidence Interval for Mean		Minimum	Maximum	Regression		ICC	*P*-value
										r	P-value		
						Lower Bound	Upper Bound						
Axial length	Low-quality	IOLMaster700	60	24.22	2.65	23.53	24.90	21.15	34.47	0.999	<0.001	0.999	0.001*
		Ultrasound	60	24.18	2.63	23.50	24.86	21.05	33.92				
	Moderate quality	IOLMaster700	119	23.79	1.80	23.46	24.12	20.46	31.35	0.999	<0.001	0.999	<0.001*
		Ultrasound	119	23.74	1.80	23.42	24.07	20.37	31.30				
	High-quality	IOLMaster700	60	23.26	1.21	22.95	23.57	20.63	27.12	0.999	<0.001	0.999	0.001⁎⁎
		Ultrasound	60	23.23	1.19	22.92	23.53	20.61	27.02				
	Total	IOLMaster700	239	23.76	1.95	23.52	24.01	20.46	34.47	0.999	<0.001	0.999	<0.001*
		Ultrasound	239	23.72	1.94	23.48	23.97	20.37	33.92				

⁎ Wilcoxon Signed Ranks Test.

⁎⁎ Paired samples *t*-test. N, Number; ICC, intraclass correlation coefficient; AL, axial length.

AL, Axial length.

**Fig. 3:**
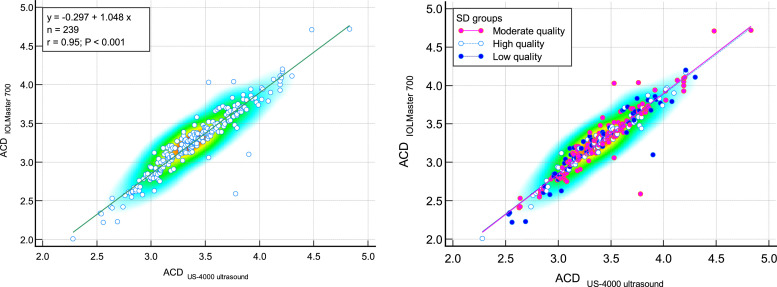
Scatter plots to compare mean values of anterior chamber depth (ACD) measured with IOLMaster700 optical biometer (Carl Zeiss Meditec AG, Jena, Germany) and Echoscan US-4000 ultrasound biometer (Nidek Inc, Japan) among all patients (left), and patients classified by the quality of measurements (right). The solid line shows the regression line.

**Fig. 4:**
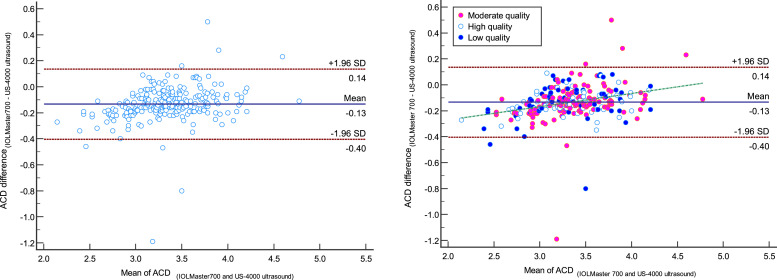
Bland–Altman plot for anterior chamber depth (ACD) comparing IOLMaster700 optical biometer (Carl Zeiss Meditec AG, Jena, Germany) vs Echosccan US-4000 ultrasound biometer (Nidek Inc, Japan) among all patients (left), and patients classified by the quality of measurements (right). The dashed green line shows the regression line.

The comparison of measured ACD by IOLMaster700 optical biometer and Echoscan US-4000 ultrasound biometer in different measurement quality of AL are reported in Table 3. Scatter plots to compare ACD measured with IOLMaster 700 optical biometer and US-4000 ultrasound biometer among all patients are presented in Fig. 3. Also, Bland–Altman plots for ACD comparing IOLMaster 700 optical biometer versus the Echoscan US-4000 ultrasound biometer among all patients are shown in Fig. 4.

3. ***The comparison of lens thickness between two devices***

**Table 3:** The comparison of measured anterior chamber depth with IOLMaster700 optical biometer (Carl Zeiss Meditec AG, Jena, Germany) and US-4000 ultrasound biometer (Nidek Echo Scan, Japan) in different measurement quality of axial length.

	Measurement quality	Devices	N	Mean	Std. Deviation	95% Confidence Interval for Mean		Minimum	Maximum	Regression		ICC	*P*-value
						Lower Bound	Upper Bound			r	P-value		
ACD	Low-quality	IOLMaster700	60	3.21	0.45	3.09	3.33	2.22	4.20	0.948	<0.001	0.941	<0.001*
		Ultrasound	60	3.34	0.41	3.24	3.45	2.53	4.30				
	Moderate quality	IOLMaster700	119	3.32	0.43	3.24	3.39	2.41	4.72	0.933	<0.001	0.927	<0.001⁎⁎
		Ultrasound	119	3.45	0.38	3.38	3.52	2.63	4.83				
	High-quality	IOLMaster700	60	3.25	0.42	3.14	3.36	2.01	4.17	0.979	<0.001	0.977	<0.001*
		Ultrasound	60	3.39	0.40	3.29	3.49	2.28	4.21				
	Total	IOLMaster700	239	3.27	0.43	3.22	3.33	2.01	4.72	0.949	<0.001	0.945	<0.001*
		Ultrasound	239	3.41	0.39	3.36	3.46	2.28	4.83				

⁎ Paired samples *t*-test.

⁎⁎ Wilcoxon Signed Ranks Test. N, Number; ICC, intraclass correlation coefficient; ACD, anterior chamber depth*.*

ACD, anterior chamber depth.

ACD, anterior chamber depth.

**Fig. 5:**
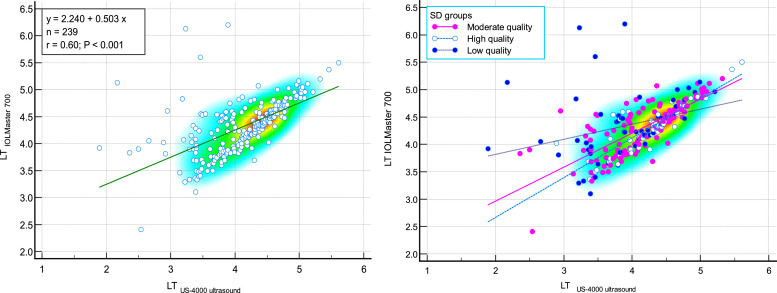
Scatter plots to compare mean values of lens thickness (LT) measured with IOLMaster700 optical biometer (Carl Zeiss Meditec AG, Jena, Germany) and Echoscan US-4000 ultrasound biometer (Nidek Inc, Japan) among all patients (left), and patients classified by the quality of measurements (right). The solid and dashed lines show the regression lines.

**Fig. 6:**
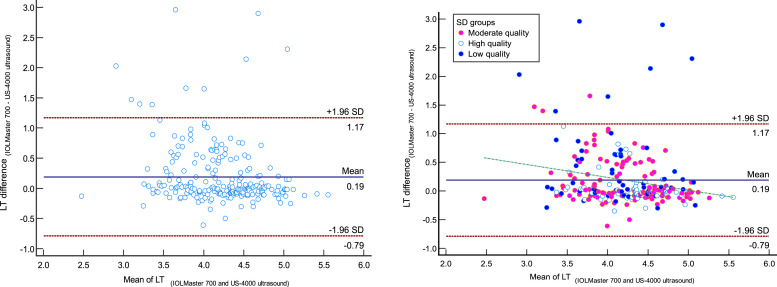
Bland–Altman plot for lens thickness (LT) comparing IOLMaster700 optical biometer (Carl Zeiss Meditec AG, Jena, Germany) vs US-4000 ultrasound biometer (Nidek Echo Scan, Japan) among all patients (left) and patients classified by the quality of measurements (right). The dashed green line shows the regression line.

The comparison of measured LT by IOLMaster 700 optical biometer and Echoscan US-4000 ultrasound biometer in different measurement quality of LT were reported in Table 4. Scatter plots to compare LT measured with IOLMaster 700 optical biometer and US-4000 ultrasound biometer among all patients are presented in Fig. 5. Also, Bland–Altman plots for LT comparing IOLMaster 700 optical biometer versus the Echoscan US-4000 ultrasound biometer among all patients are shown in Fig. 6.

**Table 4:** The comparison of measured lens thickness by IOLMaster700 optical biometer (Carl Zeiss Meditec AG, Jena, Germany) and US-4000 ultrasound biometer (Nidek Echo Scan, Japan) in different measurement qualities of axial length.

	Measurement quality	Devices	N	Mean	Std. Deviation	Std. Error	95% Confidence Interval for Mean		Minimum	Maximum	Regression		ICC	*P*-value
							Lower Bound	Upper Bound			r	P-value		
LT	Low-quality	IOLMaster700	60	4.35	0.60	0.08	4.20	4.51	3.10	6.20	0.307	0.017	0.305	<0.001⁎
		Ultrasound	60	3.94	0.67	0.09	3.76	4.11	1.89	5.21				
	Moderate quality	IOLMaster700	119	4.29	0.48	0.04	4.20	4.38	2.41	5.20	0.756	<0.001	0.742	<0.001⁎
		Ultrasound	119	4.14	0.59	0.05	4.03	4.24	2.36	5.32				
	High-quality	IOLMaster700	60	4.32	0.43	0.06	4.21	4.44	3.36	5.50	0.854	<0.001	0.843	0.239⁎⁎
		Ultrasound	60	4.28	0.51	0.07	4.15	4.41	2.89	5.61				
	Total	IOLMaster700	239	4.31	0.50	0.03	4.25	4.38	2.41	6.20	0.603	<0.001	0.593	<0.001⁎
		Ultrasound	239	4.12	0.60	0.04	4.05	4.20	1.89	5.61				

⁎ Wilcoxon Signed Ranks Test.

⁎⁎ Paired samples *t*-test. N, Number; ICC, intraclass correlation coefficient; LT, lens thickness.

LT, lens thickness.

LT, lens thickness.

In this study, all of our patients had green indicators, the SD of AL of only 9 cases was less than five μm. The SD of measured AL in our study in the short, normal, and long eyes was reported in Appendix Table 1. This table confirms that longer eyes had higher SD in both IOLMaster 700 and US-4000 ultrasound. Also, The SD of measured AL in patients with shallow, normal, and deep ACD were shown in Appendix Table 2.

**Appendix Table 1:** The SD of measured axial length in patients with short, normal, and long eyes.

AL groups	Device	Number of cases	Mean ± SD	Minimum	Maximum
Short eyes (AL < 21.5 mm)	IOLMaster 700	13	10.2 ± 5.7	4.00	21.00
	US-4000 ultrasound	13	0.020 ± 0.01	0.01	0.03
Normal eyes (21.5 to <24.50 mm)	IOLMaster 700	172	11.5 ± 6.3	4.00	46.00
	US-4000 ultrasound	172	0.025 ± 0.03	0.00	0.30
Long eyes (>24.50 mm)	IOLMaster 700	54	13.6 ± 7.0	6.00	32.00
	US-4000 ultrasound	54	.030 ± 0.02	.01	0.04

AL; axial length.

**Appendix Table 2:** The SD of measured axial length in patients with shallow, normal, and deep anterior chamber depth.

	Device	Number of cases	Mean ± SD	Minimum	Maximum
Shallow ACD (< 3.00 mm)	IOLMaster 700	58	11.6 ± 0.6	4.00	26.00
	US-4000 ultrasound	58	0.026± 0.01	0.01	0.04
Normal ACD (3.00 to <3.60 mm)	IOLMaster 700	127	11.9 ± 6.3	4.00	46.00
	US-4000 ultrasound	127	0.028± 0.03	0.01	0.30
Deep ACD (>3.60 mm)	IOLMaster 700	54	12.7 ± 7.1	6.00	32.00
	US-4000 ultrasound	54	0.032 ± 0.04	0.00	0.30

ACD; anterior chamber depth.

## Discussion

The measurement of oculometric parameters is fundamental in the preoperative evaluations before cataract surgery to determine the IOL power.^6^ The main purpose of this study was to compare the reliability and agreement between an advanced optical-based biometry machine (IOLMaster 700) and an ocular ultrasound-based biometry device (Echoscan US-4000) for measuring AL, ACD, and LT, in the groups of patients with different quality measurements.

The previous generation of the optical biometer of Zeiss company (IOLMaster 500) used a signal-to-noise (SNR) ratio to check the measurement quality. But in IOLMaster 700, the SD of the measured parameters are suggested to check the measurement quality. Based on the provided explanation by the user manual, SD values of several individual measurements in AL, ACD, and LT were 5, 7, and 6 μm. The “Quality check” dialog window also is another method that allows the user to evaluate the quality of the measurement and a green indicator indicates that the signal quality is acceptable. However, the company reported that A green signal quality indicator is no guarantee for the accuracy and reliability of the measurement. This is the first study in this field, and there is no valid classification for quality measurements. As AL is the most important measurement in ocular biometry; therefore, we decided to classify our patients based on the SD of measured AL.

In this study, we found a weak but significant correlation between SD of AL and CDVA, which could be expected on the grounds of the disturbances of the opacities (leading to lower CDVA) on the optical scan.

In terms of comparison of measured AL by IOLMaster700 optical biometer and Echoscan US-4000 ultrasound biometer, although we found significant differences in different measurement qualities of AL, these differences were equal or less than 0.04 mm.

Also, Measured ACD by two biometers had a significant difference in different measurement quality. But the comparison of measured LT by two devices showed significant differences only in low and moderate quality groups. Analysis of ICC showed that there was excellent reliability in measuring AL and ACD in different quality measurements between IOLMaster700 and Echoscan US-4000; however, there was poor, moderate, and good reliability in measuring LT in low-, moderate-, and high-quality measurements, respectively. The order of ICC values regarding the measured parameters was: AL>ACD>LT.

AL and ACD in all quality measurements showed a very strong correlation between devices with highly statistically significant. But in LT, the correlation between devices was weak with no statistically significant in low quality measurements, strong in medium, and very strong in high-quality of measurements. These findings reveal that only in the high quality measurements group, measured LT might be used interchangeably between two devices because of very strong correlation and good reliability.

Analysis of Bland-Altman plots revealed that for AL, the bias line was close to zero (0.04 mm). In our study, as shown in Fig. 2, 95% limits of agreement for AL difference were between 0.23 and −0.15 mm, and in 11 patients, measured AL showed a difference of more than 0.20 mm. It should be mentioned that the repeatability of ultrasound measurement was reported 0.11 mm.^35^ It points to the highest proportion of AL differences would be due to the low AL repeatability of ultrasound. The other important thing is related to the cooperation of patients. In this study, 11 patients with AL difference>0.2 mm were significantly older than 228 patients with AL difference<0.2 mm. It indicates that patients with AL difference>0.2 mm may have poorer cooperation because of higher age. Therefore, in addition to the low AL repeatability of ultrasound, the cooperation of patients is another probable reason for the AL difference>0.2 mm.

The Bland-Altmann plot of ACD reveals a positive correlation between differences and means that the deeper the ACD, the higher the difference between IOLMaster and echoscan. But based on the obtained results from Fig. 4, it is clear that in ACD=3.30, the mean difference is equal to zero, which means the highest chance of interchangeability of two devices, but the lower or higher than this ACD value, the mean difference increased. In patients with shallow AC, the probable causes of these differences would be that measured ACD by ultrasound affected by the pressure, and in patients with deep AC, measured ACD had higher SD and, consequently, lower measurement accuracy. However, further research is needed to find the exact cause of these differences.

The Bland-Altmann plot of LT shows a negative correlation between differences and means, in the sense that thicker LT led to less difference between the two devices (with IOLMaster being "thicker"). As the ultrasound technique should not affect LT, these progressive differences would be related to the effects of refractive indices or uncertainty to the optical measurement.

Although the accuracy of optical biometry devices is higher than ocular ultrasound, this equipment is expensive and may have limitations in measuring ocular biometric data in patients with dense cataracts.^36^ On the other hand, ocular ultrasound biometry is still a frequently used technique for measuring AL and IOL power calculation in most developing countries that is due to its lower cost and higher familiarity with the method compared to optical biometry devices.^28^ Therefore, it could be helpful for practitioners to know the interchangeability of optical biometry devices with ultrasound biometry devices. Most of the previous studies on IOLMaster700 have compared its measurements with other optical biometry devices.^11^^,^^37^, ^38^, ^39^ However, there are very few published studies regarding the measurements of IOLMaster 700 compared with an ultrasound-based biometer.^24^ These studies found similar or slightly better results for optical biometry devices.^40^^,^^41^ Cho et al. compared the biometry parameters of IOLMaster700 with five different biometry devices, including an ultrasound biometer (Pacscan 300A; Sonomed Inc., Chicago, IL, USA).^24^ They found that AL data of IOLMaster700 and ultrasound biometry could be applied interchangeably; however, ACD values could not be interchangeable. In comparison to their study, we compared the outcomes of IOLMaster 700 and Nidek US-4000, and we found that the mean value of AL and ACD, in all categories of measurement quality using IOLMaster700 can be used interchangeably by Echoscan US-4000 ultrasound biometry device. However, although the AL and ACD data showed very strong correlations and excellent reliability (AL: *r* = 1.0, *P* < 0.001, and ACD: *r* = 0.95, *P*<0.001), LT values demonstrated moderate correlation and moderate reliability in all patients who were examined through IOLMaster 700 and Echoscan US-4000 (*r* = 0.6, *P*<0.001). In a prospective study, Nakhli investigated the relationship between the measured AL with optical biometry and ultrasound in 55 consecutive patients (68 eyes) who were referred for cataract surgery.^28^ Consistent with our study design, AL in each eye was measured once by optical biometry and once by ultrasound, and the agreement between devices was evaluated. Based on the obtained results, there was strong reproducibility (99.4%) and agreement (*r* = 0.987) between both devices (*P* < 0.001). They concluded that AL measurements with optical biometry and ultrasound are well related. Although we also found the same results regarding AL measurements with both devices, in this study, Echoscan Nidek US-4000 was used, which was a different ultrasound biometry machine. In a study conducted by Rose and his coworkers, a comparison of AL measured using applanation A-mode applanation ultrasound and Zeiss IOLMaster biometer optical system was made.^13^ This cross-sectional study was performed on 51 eyes of 46 patients referred for cataract surgery. On average, the AL measured by IOLMaster was 0.15 mm higher (>0.01) compared to ultrasound biometry. We also found that the mean values of AL measured by IOLMaster was only 0.04 mm higher than measurements of Echoscan US-4000 (*P* = 0.819). The authors concluded that IOLMaster 700 provides accurate AL measurement of the eye, and it is quick and easy to use, provides no contact and no risk of infection or corneal damage. Although in their study, the optical biometer IOLMaster made by Zeiss company was used, but its model was an old version.

AL measurement results with previous version of IOLMaster (IOLMaster 500) could be easily evaluated by numerical values ​​called the reliability factor with the SNR of the measured waveform. The value of SNR obtained by IOLMaster 500 can reflect the accuracy of the AL measurement.^20^ Instead, IOLMaster 700 uses a new metrics known as SD factor for checking quality of its measurements. In a retrospective chart review, Tao Ming et al. compared measurements of optical and ultrasonic biometric devices in patients with borderline SNR.^42^ They aimed to determine whether optical and ultrasonic biometric measurement of AL and IOL power in cases with borderline SNR has a significant difference compared to ultrasound biometry. Sixty patients with cataracts and IOLMaster biometry with borderline SNR (2.0–1.6) were included in their study. A retrospective review was conducted to compare the data collected by optical biometry and ultrasound biometry in cataract cases with borderline SNR. The results showed that optical biometric measurements of IOL and AL have no significant difference from ultrasound measurements. The analysis also showed a good agreement between these two methods. However, in their study, two different biometry tools were used; IOLMaster (IOLMaster 500, Carl Zeiss Meditec) and ultrasound biometry (Eyecubed, Ellex Inc., Minneapolis, MN, and Dublin, CA, USA), which are old versions of biometric devices. The authors suggested that in patients with borderline quality measurements, IOL power and AL measurements with optical biometry are still useful in surgical planning and that additional ultrasonography measurement may be used more as a corroborative tool. Our results also confirm their conclusion, as we found excellent reliability and agreement between the two devices in all quality measurements with both optical and ultrasound biometry devices. However, there is no report of measurements with IOLMaster 700 at different indices for quality measurement.

This work suffers from some limitations, notably related to the performing this study on two biometry devices. In addition, we did not categorize patients according to the cataract type and degree. Therefore, it is recommended that other researchers perform a similar study in normal populations as well as in cataractous patients with categorized cataract type and degree. Although the current study was conducted on two of the most common biometric devices, the results may not apply to other methods of biometric devices. Another important limitation was the absence of an interexaminer repeatability analysis. We recommend researchers formulate the mathematical correlation of AL, ACD, and LT between the optical and ultrasound biometers in different AL groups, such as short, normal, and long eyes. Also, evaluating the probable interchangeability of AL, ACD, and LT measured by the optical and ultrasound biometers in different AL groups is recommended for future studies.

## Conclusions

The results in the present study indicate that AL and ACD measured by IOLMaster700 optical biometer and US-4000 ultrasound biometer were well-related in different measurement qualities of AL when we observed green indicators on the printout of IOLMaster 700. However, only in high quality of measurements group, measured LT might be used interchangeably between two devices because of very strong correlation and good reliability. Therefore, measuring AL and ACD of the IOLMaster700 optical biometer can be used interchangeably with US 4000-ultrasound biometer in different quality measurements. In other words, the reliability of the AL and ACD measurements with the IOLMaster700 was not influenced by the differences in measurement quality. Nevertheless, LT should be used interchangeably cautiously only in patients with high-quality measurements.

## Conflicts of Interest

The author has no conflicts of interest to declare.
